# Supplementary material for: Identifying Predictors Associated with Risk of Death or Admission to Intensive Care Unit in Internal Medicine Patients with Sepsis: A Comparison of Statistical Models and Machine Learning Algorithms
Source: Antibiotics (Basel). 2023 May 18;12(5):925. doi: 10.3390/antibiotics12050925 (PMC10215570; doi:10.3390/antibiotics12050925)
Supplement: Supplementary file 1 [file antibiotics-12-00925-s001.zip › antibiotics-2375767-supplementary.pdf]

Figure S1. Plot of the 10-fold cross-validation curve of the mean errors (binomial deviance) made by different multivariable LASSO models (red dotted line) estimated at varying penalty parameters (λ), along with the upper and lower standard deviation around the estimates (error bars).

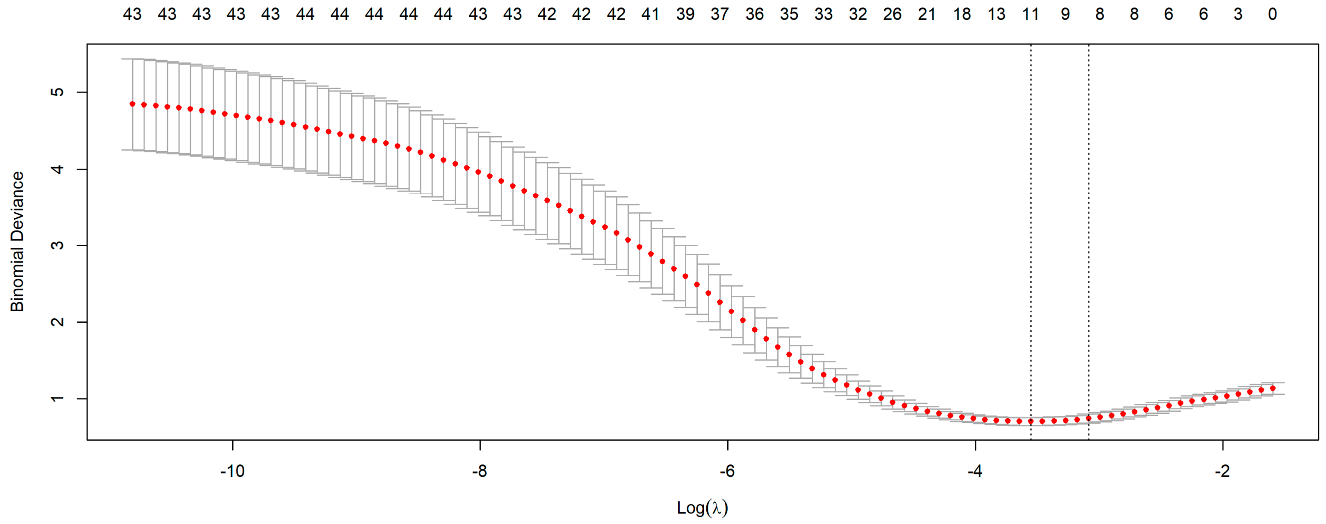

Two special values along the λ sequence (log-values) are indicated by the vertical dotted lines. The leftmost λ value provides the minimum mean cross-validated error, while the rightmost λ value provides the most regularized model such that the cross-validated error is within one standard error of the minimum. The axis above indicates the number of nonzero coefficients detected at the current λ.

Figure S2. Plot of the 10-fold cross-validated mean relative error made by the tree at varying values of the complexity parameter (cp), along with the upper and lower standard error around the estimates (error bars).

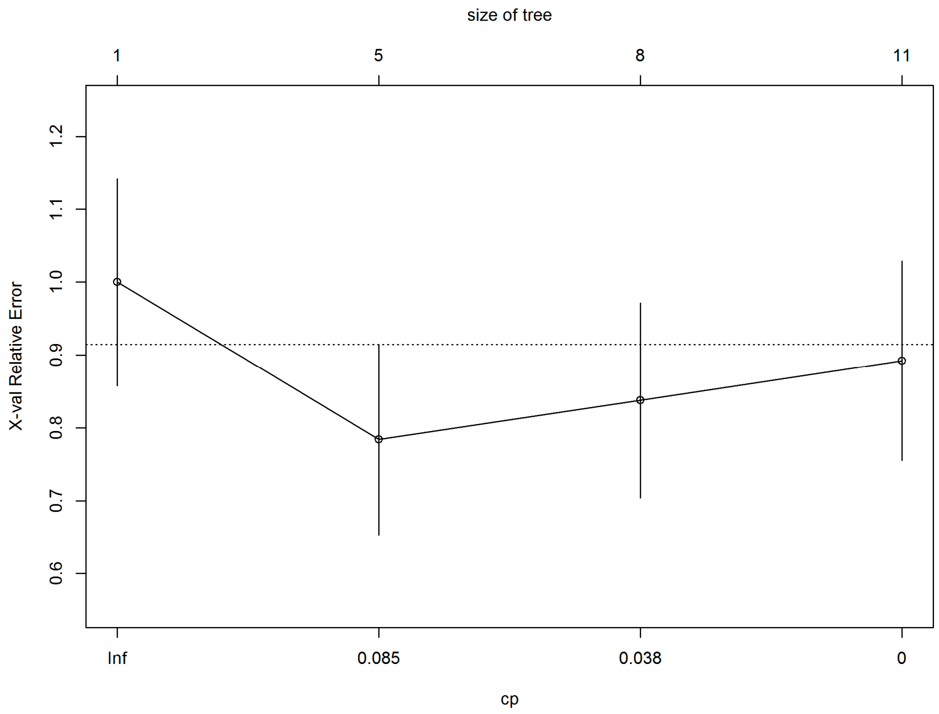

The complexity parameter is used to control the size of the decision tree and to select the optimal tree size. When cp=0 a tree with its maximum depth is built (i.e. large tree). The axis above indicates the number of terminal nodes of the tree at the current cp. The minimum cross-validation error was reached at cp=0.085 and this returned a pruned final tree with 5 terminal nodes. The horizontal dotted line is drawn at 1 standard error above the minimum of the curve (i.e. at cp=0.085).
